# COMPUTING, DENTISTRY AND AGING: ORAL HEALTH, BIOLOGICAL AGE AND MORTALITY

**DOI:** 10.1093/geroni/igac059.3092

**Published:** 2022-12-20

**Authors:** Xin Zhong

**Affiliations:** A*STAR Singapore, Singapore, Singapore

## Abstract

Oral health is essential to general well-being across the lifespan. Recent study on number of natural teeth, denture use and mortality in non-Singaporean elderly indicated the importance of oral determinants. To the best of our knowledge, no study has envisioned the association between biological age and oral health among community-dwelling older Singaporeans. By leveraging the acquired knowledge from Singapore Longitudinal Ageing Study (SLAS), our aim is to examine the association between biological age, oral health, and mortality. Our hypothesis is that older adults with or without dental problems will have a significant difference in mortality and other health outcomes. Our methodology is to combine a more realistic AI approach and a hypothesis-driven multidisciplinary approach to examine the association between oral health and general health outcomes (mortality, hospitalization, etc). Cross-sectional data analyses were performed on 2844 community-living Chinese older adults of 65–80 years old in the Singapore Longitudinal Aging Study II (SLASII) cohort. The Keplan-Meier survival analysis showed statistically significant differences in survival time between the group with dental problems and the group without dental problems (p < 0.001) in an 8-year longitudinal study. Results also showed a significant difference between biologically younger, biologically older, and the intermediate group groups (p = 0.0012). Those groups were classified by the computed biological age. Our preliminary results will produce a plethora of new links between oral health, biological age, and mortality. The research outcome may serve as a baseline for policymakers to evaluate and promote subsidized oral health programs.

